# Brazilian Citizens’ Opinions and Attitudes about Farm Animal Production Systems

**DOI:** 10.3390/ani7100075

**Published:** 2017-09-28

**Authors:** Maria C. Yunes, Marina A. G. von Keyserlingk, Maria J. Hötzel

**Affiliations:** 1 Laboratório de Etologia Aplicada e Bem-Estar Animal, Departamento de Zootecnia e Desenvolvimento Rural, Universidade Federal de Santa Catarina, Florianópolis 88034-001, Brazil; mcyunes@hotmail.com; 2 Animal Welfare Program, Faculty of Land and Food Systems, The University of British Columbia, Vancouver, BC V6T 1Z4, Canada; nina@mail.ubc.ca

**Keywords:** animal welfare, ethics, livestock production, qualitative research, survey

## Abstract

**Simple Summary:**

The inclusion of societal input is needed for food animal production industries to retain their “social license to operate”. Little is known about the knowledge and attitudes of Brazilian citizens regarding food animal production systems. The aim of this study was to explore the beliefs and attitudes of Brazilians not associated with livestock production towards farm animal production systems. Overall, our participants expressed a preference for free-range, cage-free, and more “natural” production systems. They also expressed concerns with livestock production systems that limited the movement or expression of natural behaviours, particularly those that they associated with animal suffering or distress. They recognized farm animals as deserving respect and dignity beyond the provision of basic needs. Our findings indicate that Brazil’s current farm animal housing practices that are associated with restriction of movement may not align with societal expectations.

**Abstract:**

The inclusion of societal input is needed for food animal production industries to retain their “social license to operate”; failure to engage with the public on this topic risks the long-term sustainability of these industries. The primary aim of this study was to explore the beliefs and attitudes of Brazilians citizens not associated with livestock production towards farm animal production. A related secondary aim was to identify the specific beliefs and attitudes towards systems that are associated with restriction of movement. Each participant was shown pictures representing two of five possible major food animal industries (laying hens, beef cattle, pregnant sows, lactating sows, and poultry meat). Participants were presented a six pages survey that included demographic questions plus two sets of two pictures and a series of questions pertaining to the pictures. Each set of pictures represented a particular industry where one picture represented a housing type that is associated with behavioural restrictions and the other picture represented a system that allowed for a greater degree of movement. Participants were asked their perceptions on the prevalence of each system in Brazil, then their preference of one picture vs. the other, and the reasons justifying their preference. Immediately following, the participant repeated the same exercise with the second set of two pictures representing another industry followed by the same series of questions as described above. Quantitative data were analysed with mixed effects logistic regression, and qualitative responses were coded into themes. The proportion of participants that believed animals are reared in confinement varied by animal production type: 23% (beef cattle), 82% (poultry), 81% (laying hens), and 60% (swine). A large majority (79%) stated that farm animals are not well-treated in Brazil. Overall, participants preferred systems that were not associated with behavioural restriction. The preference for free-range or cage-free systems was justified based on the following reasons: naturalness, animals’ freedom to move, and ethics. A minority of participants indicated a preference for more restrictive systems, citing reasons associated with food security and food safety, increased productivity and hygiene. Our results suggest that the majority of our participants, preferred farm animal production systems that provide greater freedom of movement, which aligned with their perception that these systems are better for the animal. Our results provide some evidence that the current farm animal housing practices that are associated with restriction of movement, which are gaining traction in Brazil, may not align with societal expectations.

## 1. Introduction

In many developed and developing countries, familiarity and knowledge about farm animal production systems has decreased among the general public due in part to the growing distance between locations where agriculture practices take place and where the majority of consumers live [1,2,3]. In addition to urbanization, some also argue that media and advertisements [4] that reinforce the historical romantic view of agriculture [1] also contribute to the growing lack of knowledge of modern agricultural production practices. This disconnect may explain why the lay public, when confronted with the realities of the intensive animal industries, frequently express negative attitudes towards them [5,6,7,8]. 

Livestock production practices vary widely, with specific practices viewed by the public as more or less favourable depending on what aspect is being questioned. For instance, the main animal welfare concerns raised by the public regarding the use of confinement (no outdoor access) and cage systems for farm animals are that they prevent animals from moving freely, provide inappropriate social contact, and are frequently associated with a barren environment with no outdoor access [9,10,11,12,13,14]. European citizens identified caged hens, broilers and pigs as the top three farm animals they believed to have the poorest welfare [9]. Similarly, New Zealand citizens [15] specifically identified these three farm animals as requiring the most improvement in terms of animal welfare standards and in need of legal protection. 

Farm animal welfare issues have often been viewed to be a consequence of conflict between producers and citizens/consumers. Citizens’ attitudes towards different food production systems are not only dependent on rational assessment of risks, benefits, economics, and nutrition, but also reflect ethical and moral considerations [16,17]. Positive responses from farm animal producers and retailers to society’s concerns about controversial agricultural practices have led to some changes in livestock production practices in several countries [18]. Policy makers and industry stakeholders in some countries have also begun to consult the public in the process of defining acceptable livestock welfare standards [19].

It has been argued that Brazilian lay citizens have little knowledge of animal production systems [20,21]. However, lack of knowledge does not mean that they have no concerns or negative attitudes toward current animal production practices and systems. For example, urban Brazilians were reported to be concerned with food quality, view food additives, hormones and pesticides as hazards [8,22,23] and have negative attitudes towards genetically-modified food, based on perceptions of risk and lack of naturalness [24,25]. Several studies on public views of farm animals production systems have been undertaken in Europe and North America [10,26,27,28,29,30]. This issue, however, has received considerably less attention in Brazil, a leading food animal producing country and a large urban consumer market [31,32].

The primary aim of this study was to explore the beliefs and attitudes of Brazilians not associated with livestock production towards farm animal production, and the underlying reasons. A secondary aim was to identify the specific beliefs and attitudes towards systems that are associated with restriction of movement. 

## 2. Materials and Methods

This study was approved by Ethics Committee on Experimentation of the Santa Catarina State University 1.111.587 (22/06/2015).

### 2.1. Participants’ Recruitment

Participants were recruited exclusively through direct approach at locations known to be associated with intense movement of people or waiting times, such as technical and scientific events and fairs, the local airport and interstate bus terminal in Florianópolis, Santa Catarina. These venues provided the opportunity to identify participants of both sexes, of different ages and geographic backgrounds. To increase the diversity of public opinions we tried, whenever possible, to achieve a balanced distribution between participants’ sex and ages. 

Participants were approached randomly and asked to voluntarily participate in the survey. Conditions to participate in the research were that the participant was at least 18 years old, a Brazilian citizen and was available and interested in voluntarily answering a short questionnaire covering the general theme “animal production”. Each participant received a consent form that they were asked to read and if they agreed to participate were required to sign before they began the survey that was 6 pages long and included a total of 19 questions and two sets of two images. The identity of the participants was not required.

### 2.2. Description of the Survey

Data collection was conducted during the months of September 2014 to June 2015. 

The first questions addressed participants’ socio-demographic information relating to sex (male, female), age (18–25, 26–35, 36–45, 46–55, 56–65, over 66 years), education (elementary school, high school, or higher education), the region of Brazil which they viewed to be their primary residence (south, southeast, north, northeast, midwest), whether they lived in a rural area, small town (up to 20,000 inhabitants), medium town (from 20,000 a 100,000 inhabitants), large city (more than 100,000 inhabitants), or metropolis (more than one million inhabitants), if they had ever lived outside the country (yes, no), and their level of association with livestock production (“not associated”–no ties with the animal industries–or “associated”–any ties with the animal industries such as veterinarian, livestock production professional, consultant/manager, producer, student or faculty in any field of animal agriculture). Participants were also asked how informed they considered themselves to be regarding animal production (very informed, somewhat informed, intermediate, somewhat uninformed, totally uninformed); their main sources of information about raising animals used for food production (multiple choice: internet, TV or radio (general programs), TV or radio (rural programs), newspaper (printed or electronic), specialized magazines, animal protection society websites, university, friends, other); if they consumed animal products (yes, no); if they considered farm animals in Brazil to be well-treated (yes, no); and finally, how much they cared about the quality of life of the animals used in food production (very, intermediate, not at all).

**Figure 1:**
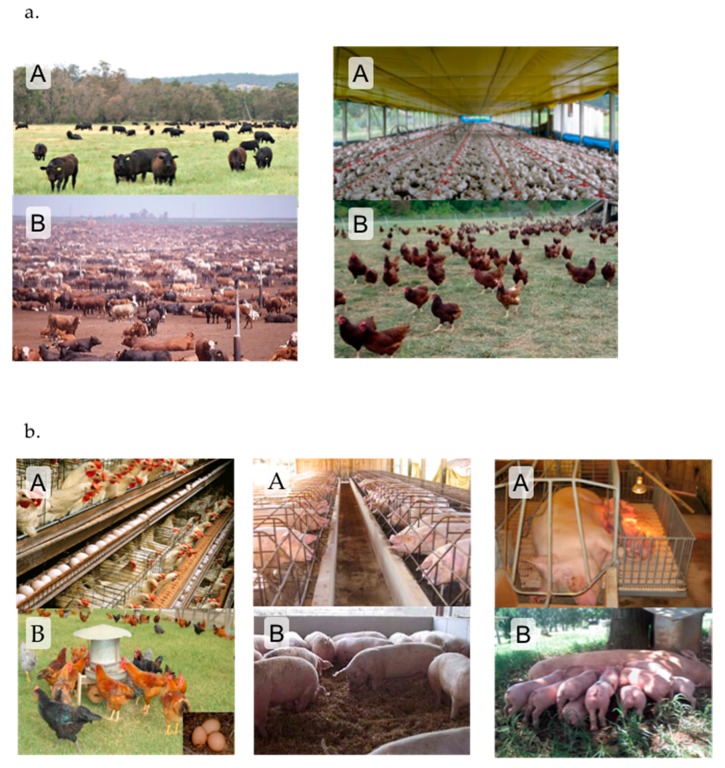
Each respondent were presented with a series of photos (**a**) one example of a non-confined system and a confined cage-free system for the same species (either beef cattle or poultry), and (**b**) one example of a cage-free system and a caged system for a second species (either laying hens, gestating sows, or farrowing sows). The order of the photos was randomized so that each set of paired photos appeared 50% of the time either as the first or second. In each case respondents were asked “which situation (A or B) would you like to be the more common in animal production in Brazil?”

Two pages with two images each were presented to each participant but separated by three questions that were repeated after each set of pictures. Each page showed a set of two images showing the same species but in two different production systems: free-range beef cattle and beef cattle in intensive open-air confinement (feedlot); free-range broilers or broilers in intensive indoor confinement; free-range laying hens or layers in battery cages; free-range farrowing sows or sows in farrowing cages; and, group housed gestating sows or gestating sows in individual cages. Each respondent compared one example of a non-confined system with a confined cage-free system for the same species (either beef cattle or poultry, Figure 1a) and one example of cage-free system with a caged system for a second species (either laying hens, gestating sows, or farrowing sows, Figure 1b). The order of the images was randomized so that each set of paired images (cases) appeared 50% of the time either as the first or second. After each set of paired images the respondent was asked to indicate their knowledge of the prevalence of these systems (“In your opinion, which of these situations is the most common in commercial production in Brazil?” and offered as answer with choices “A”, “B”, “both are equally common in Brazil”, “neither is common in Brazil” and “I do not know”). The respondent was then asked to indicate their preference for one of the two systems (“Which situation would you like to be the more common in animal production in Brazil?” with the answer options “A” and “B”). Finally, the respondent was asked to justify their preference with an open answer (“Please justify briefly why you prefer the livestock system you indicated above”). 

The entire questionnaire was initially tested using 20 randomly recruited participants, with their responses used to refine the questionnaire prior to release. The images used in the questionnaire were also tested to ensure that they represented the issue we intended to address and avoid examples that participants may have considered “extreme” of a given situation. To ensure that images followed this criterion, six people (three experts in animal production and three lay people) were consulted. Images that were considered to convey a typical example of a given production system were selected for use. After the initial testing, we concluded that presenting two cases yielded the most detailed answers and justifications. In contrast, when we provided three or more cases many respondents answered subsequent questions with phrases such as “same as previous”, or “same reason”.

### 2.3. Data Analyses

Approximately 100 responses were collected for each case, totalling 612 questionnaires, which were considered complete if the respondent completed the entire questionnaire. Participants with any level of association with livestock production were excluded from the current analysis, resulting in a final sample of 479 completed questionnaires. 

To analyze the quantitative data, we used a mixed effects logistic regression that was fitted accounting for the random effect of region. Two models were fitted: one assessed respondents' preference for cage or cage-free systems, and the other the preference for confined or free-range systems as response variables. Explanatory variables were screened using univariable analysis, with variable having a *p* < 0.2 included in the final multivariable models. Models were reduced using manual stepwise backward elimination using a *p* < 0.05 as threshold for keeping the predictors in the model. Explanatory variables tested were: sex, age, education, rural or urban living, how informed respondents considered themselves to be regarding animal production, if they considered farm animals in Brazil to be well-treated, and how much they cared about the quality of life of the animals used in food production. Logistic regressions were fitted using lme4 package [33] on R [34].

Open answers were analysed using qualitative analyses, based on the method described by Huberman [35], which has three stages completed in the following order: *data reduction* (information is coded finding themes), *data display* (organization of the information permitting to reach conclusions) and *conclusion drawing and verification* (noting of patterns and themes and using confirmatory tactics such as triangulation between three readers). To ensure that the coding of themes was appropriate to the proposed objectives, and therefore valid (i.e., that it represented all content displayed on the information collected), first three readers analysed 20 random responses, turning them into codes used to identify themes. The three readers then compared their results and discussed any discrepancies and ambiguities until agreement was reached. Two readers then coded the first 100 answers to ensure agreement. From that point on the lead author undertook the remaining encodings with the codes organized, counted and grouped into major themes. 

Twelve codes were identified from the responses presented by participants to justify their preference for a given image within a case, which were grouped into four themes: “animal welfare”, “production”, “product quality” and “environmental issues”. The theme “animal welfare” comprised codes related to the quality of life of animals: freedom (including aspects such as the ability to move and issues related to amount of space provided to the animal or movement), natural life (related to expression of natural behaviours and the natural habitat of the animal), sentience (the ability of animals to express positive and negative feelings), animal health (physical and biological), animal stress (physiological or psychological), quality of feed offered to animals, and ethics (related to the respondent’s values regarding the use of animals by humans, references to the system as “cruel” or “inhuman”, or claims of the existence of better alternatives for animal production). 

The theme “production” comprised codes related to the productive systems, including naturalness (the production system should be as natural as possible), productivity (efficiency of the system, the cost of the resulting product to consumers, or the area needed for animal production), control (referring to management, hygiene, animal health, and diseases), and ethics (participants expressed values regarding food production and food supply to the human population). The theme “product quality” included two codes: inputs used in production of food to humans and animal food (including pesticides, hormones and antibiotics), and human health (references to the influence of the resulting food product on human health). The theme “environmental issues” included possible benefits or risks of the particular production system on the environment. 

On some occasions, the topics coded under naturalness and inputs were not related to any theme, as some participants gave short answers, like “more natural” or “less hormones”; respondents did not explain if this concern referred to the animal, the system, the quality of the products, or the environment. 

Quotes were translated to English by the first and last author.

## 3. Results

The demographics of participants (*n* = 479) are reported in Table 1. We did not recruit any participant that identified himself or herself as illiterate. Six participants did not consume animal products. Most participants (79%) considered that farm animals are not well-treated in Brazil. For 39% of the participants, farm animal welfare was stated to be a major concern and for 52%, it was viewed to be of some concern. 

In terms of how informed participants considered themselves to be about animal production, 36 (7%) said very informed, 151 (31%) somewhat informed, 164 (34%) intermediate, 95 (20%) somewhat uninformed, and 36 (8%) totally uninformed. Participants indicated the following when asked specifically about their main sources of information on farm animal rearing: Internet (65%), TV and radio (38%), friends (35%), newspapers (18%), specialized magazines (14%), universities (12%), and websites of animal protection societies (9%). 

The proportion of participants that believed that farm animals in Brazil are reared in intensive confinement or caged systems varied by animal industry: 23% for beef cattle, 82% for poultry, 81% for laying hens, 56% for gestating sows, and 63% for farrowing sows. When asked about their preferred system, 87% chose free-range systems and 78% chose cage-free systems. The only variable tested that showed a significant relationship with the preference for the system was opinion regarding the quality of treatment of farm animals in Brazil: respondents who thought that animals are not well-treated in Brazil had a stronger preference (*p* < 0.05) for cage-free over the caged systems and free-range over the confined systems (OR = 3.43; 95% confidence interval: 2.10–5.59, and OR = 3.8; 95% confidence interval: 1.92–7.27, respectively).

### 3.1. Reasons to Justify the Preference for a Given Image

Examples are presented followed by the number of the respondent (R) and the image chosen within brackets. The frequency of the themes identified in the responses are summarised in Table 2.

### 3.2. Animal Welfare

The main reason offered by participants justifying their preference for free-range or cage-free systems was the animals’ freedom: *Cattle should live free on pasture* (R34 (free-range beef cattle)); *Because animals are raised with more freedom* (R320 (free-range poultry)). Freedom was also implied in the context of sufficient space for animals to walk or to move around: *More space for animals to move around* (R109 (cage-free gestating sows)). Some respondents associated freedom with lack of stress, healthier animals, and better product quality: *Animals are free, quiet in their habitat, without suffering stress* (R467 (free-range poultry)); *I think that free animals are healthier* (R67 (cage-free laying hens)); *In the chosen image animals are free, producing better eggs, and the animals themselves are in better shape* (R45 (cage-free laying hens)). 

Many participants considered that animals should have the opportunity to live either in a natural way or in their natural habitat: *it is the natural habitat of animals* (R318 (cage-free farrowing sows)); *a life closer to their natural environment may be better for the animals* (R466 (free-range poultry)). Many participants also associated a natural life with freedom from stress and better product quality: *The animals are less stressed living naturally, resulting in products of greater nutritional quality* (R338 (free-range poultry)); *I believe that farm animals raised in open environments, closer to nature, produce food of better quality, and the animals will have a better quality of life* (R78 (cage-free laying hens)); *I think it is closer to the natural habitat, causing less stress to the animals, and consequently reducing (the use of) harmful chemical inputs and mistreatment* (R449 (free-range poultry)).

Some participants argued that animals deserve respect and must be well-treated: *it is fairer to the animals and to consumers* (R445 (free-range poultry)); *I think it is more humane, more natural, and less cruel* (R338 (free-range cattle)); *Because animals do not deserve to be mistreated* (R444 (cage-free gestating sows)). 

A few participants associated confined or caged systems with good welfare, e.g., animals being well cared for, free of stress or free of diseases *better treatment and feeding* (R412 (caged gestating sows)); *An appropriate place to raise piglets* (R165 (caged farrowing sows)).

### 3.3. Product Quality Is a Desired Outcome of Livestock Production Systems

Besides perceiving an association between animal welfare and product quality, participants were concerned about the influence of the quality of food offered to the animals, the use of chemical inputs and potential residues on the food produced, and the hygiene of the system, on human health. 

The quality of the animals’ feed was a salient topic among participants that preferred free-range cattle, who emphasized the relation between animals’ feed and product quality and showed a preference for providing feed to the animals that was free of chemicals: *when we consume the product of these animals we are also ingesting what they ingested* (R392 (free-range poultry)); *The meat we eat nowadays (reflects) more concentrate feed than actual meat* (R244 (cage-free laying hens)); *The way animals are fed with chemical inputs that bring us health problem* (R72 (cage-free farrowing sows)). Many expressed concerns about the use of chemical or veterinary drug residues on their food: *Because of the excessive use of hormones and the non-organic fattening system* (R193 (cage-free farrowing sows)); *Because it is a better product for our health, with less use of substances during its production that may make us sick. And because I think they are tastier* (R290 (free-range poultry)); *Animals develop naturally, producing healthier meat* (R277 (cage-free laying hens)).

For some participants good hygiene practices were a requirement for product quality; many related caged and confined systems with better hygiene and disease control: *Because (on confined systems) diseases, hygiene can be controlled* (R300 (confined poultry)); *Apparently it (the confined system) is more hygienic, conveys greater security and confidence for human consumption* (R130 (caged farrowing sows)). 

The concern with hygiene was especially salient in the case of swine production, with caged systems often associated with better hygiene: *better vaccination care, better sanitary control* (R459 (caged gestating sows)); *Because there is more surveillance and control, and therefore, more hygiene and a healthier life* (R124 (caged gestating sows)). The same concerns with hygiene were also evident among some participants that preferred cage-free systems: *Less confinement, though I do not know how the hygiene issue would be solved* (R90 (cage-free gestating sows)); *It is obvious that the first image (cages) shows hygiene; but thinking of the animals the second image (group housing) is the best* (R90 (cage-free gestating sows)).

### 3.4. Naturalness of the Production Systems

Naturalness was an important and desired issue for participants that preferred free-range or cage-free systems. Respondents associated naturalness with freedom from suffering: *Because it is more natural and doesn’t harm the animal* (R161 (cage-free farrowing sows)); natural behaviours and natural living: *Animals should be raised in the open sky, free to be able to socialize and exercise their natural behaviours* (R427 (cage-free laying hens)); *Animals roaming free and able to express their natural behaviour* (R473 (free-range poultry)); *Because it seems more “natural”, less harmful. Animals are free to roam, similar to their natural habitat* (R93 (cage-free gestating sows)); absence of chemical inputs in the diets: *A natural production system that does not rely on drugs to accelerate production in order to achieve a fast production and commercialization cycle* (R372 (free-range poultry)); animals’ natural growth and natural development: *In this case, the animal is raised naturally and has its normal life cycle* (R53 (cage-free laying hens); *Natural cattle, without intensive fattening* (R142 (free-range cattle)); and healthier production: *In the image showing cage-free farrowing sows the system is more consistent with natural, healthier methods* (R357 (cage-free farrowing sows)).

Some participants simply expressed a preference for naturalness, e.g., *Because it is natural, and the natural process is always better* (R186 (free-range poultry)). 

### 3.5. Intensive Production for More Abundant and Cheaper Food

Some participants believed confined and caged systems can lead to higher productivity, lower costs of production, and meet consumer demand for low-cost food: *Reduces the space required to raise animals* (R423 (confined cattle)); *It allows for lower costs of production, reducing the selling price and enabling low-income people to consume this product* (R460 (confined poultry)); *Because the system on the image can meet the huge demand of the population* (R201 (caged farrowing sows)). 

### 3.6. Concerns Regarding the Environmental Impacts of Livestock Production

Some participants considered free-range and cage-free systems less aggressive to nature: *it is better for the ecosystem to have animals free in nature* (R211 (cage-free farrowing sows)); while others related intensive systems with less use of natural resources: *Confinement requires less use of natural resources, correct control of cattle health and more production in less time* (R205 (confined cattle)); *Besides using less pasture (therefore causing less deforestation), cattle are ready (for slaughter) in less time (in my lay opinion)* (R148 (confined cattle)).

## 4. Discussion

In general, our participants expressed a preference for free-range, cage-free and more “natural” production systems. Similar to reports from around the world (e.g., Europe [9,10,11,26], Canada [12,29], US [13,36,37]), participants expressed concerns with livestock production systems that they perceived to cause animal suffering or distress, limit the movement or the expression of some natural behaviours, and reduce animal health. Many participants also emphasised ethical issues related to the quality of animals’ lives, recognizing farm animals as deserving respect and dignity beyond the provision of basic needs. For some, having a good life was a requirement if the animals are destined for human consumption [26]. The similarity of our findings to studies done in other parts of world indicates that these values are common to contemporary society.

A preference for systems perceived as being more “natural” and a concern with the quality of the food produced by these systems were salient in participants’ justifications of their choices. Naturalness and better product quality were often related to production systems that do not use growth promoters and antibiotics, that feed natural food to the animals, or that allow animals to express their natural behaviours and engage in social interactions. Concerns expressed by our participants regarding the quality of food offered to the animals are in line with previous surveys with European [9,10,17,38,39] and North American citizens [13,40]. Positive values associated with naturalness in animal production and rejection of use of growth promoters and additives for animal production have both been identified in several surveys of public attitudes [8,13,14,17,41]. 

A perceived association between naturalness, animal welfare, and product quality also echoes several previous surveys done in other countries [9,13,14,42,43]. In many parts of the developed world this may be explained by abundant media coverage of cases of infectious pathogens in food, antibiotic resistance, and other “food scares”, possibly causing the public to associate food quality with good animal welfare (e.g., see [43,44]). Additionally, marketing instruments used to promote and sell animal products often rely on the use of discourses and images of naturalness that reinforce these ideals [4]. In Brazil there are additional factors that may explain this perception. For example, antibiotics are used as growth promoters in pig and poultry produced for domestic consumption [45], a practice that has recently received much attention by the Brazilian media (e.g., [46]). Additionally, there has been much debate about GMO (genetically modified organisms) food products in Brazil [47], and the data reported by the annual publication of pesticide levels (including the presence of unauthorised products) in vegetables available in the domestic market by the National Health Surveillance Agency, ANVISA [48]. There are also numerous other media reports of presence of pesticides’ residues in food [49]. Recent advertisements within the media stating that no hormones are used in domestic poultry production [50] have likely added additional confusion to this discussion, given that many individuals now question this illegal practice [51]. 

Previous studies have reported low levels of awareness among lay Brazilian citizens regarding animal production systems and practices [20,21,52]. Our survey results suggest that, though true in some cases, this degree of awareness cannot be generalised. Although most participants assessed their knowledge of livestock production systems as low and some declared total ignorance about how food animals are reared, or were brief and vague in their responses, others referred to specific practices of intensive livestock systems that were not shown in the images, such as the use of feed additives and veterinary drugs, production methods used to achieve high growth rates, as well as emphasising the importance (in their opinion) of animals expressing their natural behaviours. These statements are also supported by a recent survey of Brazilian citizens where some participants showed some awareness of specific dairy farming management practices but also views that did not resonate with some common management practices [8]. In the present survey, when asked which image they believed depicted the most common situation in Brazil, most participants seemed to be aware that the majority of pigs and poultry are reared in confined and caged systems and beef cattle on pasture [53,54]. Therefore, despite their limited knowledge of the production systems and practices, participants were able to express general expectations and criticisms regarding the quality of life of farm animals, which many associated with the quality of the food produced and thus affected human health. 

Historically, increased awareness of livestock production systems has been associated with society becoming more involved in demanding and promoting changes in livestock production systems (e.g., [55,56]). In this context, the impact of knowledge of public acceptance of animal livestock production systems has been debated in recent years. Some researchers [14,57] and farmers [58,59] assume that a more educated public will become more accepting of technologies or systems considered ideal or acceptable by animal and veterinary scientists and the associated farm animal industries. This has been discussed as a “deficit model”—in short, ignorance is the basis for a lack of societal support for issues in science and technology, and can therefore be changed with education [60]. However, it has also been discussed [42,61] that non-experts assess technologies based on risk and moral values. Indeed, numerous reports show that increasing information tends to result in increase opposition to contentious livestock production practices [8,29,40,62,63,64]. 

Although most of our participants preferred free-range and cage-free systems, some, albeit a minor proportion, indicated a preference for confined and caged systems. These participants were concerned with the impacts of the production systems on productivity and the cost of food produced, as well as the need to produce sufficient and affordable food for a growing world population [65,66]. For some participants the latter can only be achieved with caged and confined systems, which may be based on the belief that modernization and intensification are required for dramatic increases in meat production to be achieved [67]. Some of our participants also perceived advantages for the intensive systems in terms of easier animal handling and better hygiene (particularly when discussing swine production). This might be explained by the fact that Brazilian consumers perceive pork meat as a greater risk to health than beef [68]. Others have shown that, although some sensorial aspects, practicality and convenience and production following animal welfare standards are highly valued by Brazilian pork consumers, animal sanitary aspects are considered the most important [69]. Possibly due to historical and cultural images of early pork production systems that existed in the country [68,70], many Brazilians associate pork meat with a source of zoonotic diseases–especially worm infestations–and high cholesterol [71,72]. Accordingly, a Dutch survey [73] found that citizens appreciate some aspects of modern animal farming, such as good hygiene practices and technological innovations that help animal management. Indeed, several studies have reported that food safety is the highest-ranking attribute mentioned by survey participants [38,74,75,76,77]. Other reasons for Brazilians to be concerned with food safety and hygiene are the recurrent cases of milk frauds [78,79], or illegal slaughter of animals [80] that are usually conveyed with images of poor infrastructure by the media (e.g., [81]).

Finally, this qualitative, exploratory study was based on a convenience sample of participants, and as such cannot be interpreted as representing the views of the Brazilian society. In comparison to the Brazilian population [31] our sample contains a greater proportion of well-educated citizens, likely linked with citizens’ wealth. There were respondents from all five regions of the country, but with a disproportionate over representation of the south and southeast regions and under representation of the northeast region. Although we acknowledge that the sample is unbalanced in terms of socioeconomic and educational stratification, we argue that the highly educated participants represent a segment of opinion holders that have substantial purchasing power, traits that may influence changes in production practices. This survey contributes original information on an issue underexplored in developing countries [82], which are the fastest growing producers and consumers of animal food products [32]. 

## 5. Conclusions

Many practices used in intensive animal production systems seem far removed from the moral values and expectations of our sample of the Brazilian public. Participants’ showed limited awareness of animal food production systems and practices used in Brazil, but were critical of perceived outcomes of practices and systems on the quality of the products and in regards to the lives led by the animals (e.g., suffering, freedom, health), and subsequent risks to human health. Legislation [83] or retail and industry-led changes in husbandry practices that are starting to happen in developing countries like Brazil (e.g., [84,85,86,87,88]), may be costly to producers if they are required to comply with mandated requirements [56]. However, these initiatives may not be sustainable if they are implemented in the absence of dialogue with society per se [32], for example, if these changes do not meet the expectations of those demanding them [89,90]. 

## Figures and Tables

**Table 1:** Demographics of survey participants and of Brazilians according to latest Brazilian Institute of Geography and Statistics (IBGE) census [31].

Demographics	Participants *N* (%)	IBGE Census Data (%)
**Sex**		
Female	255 (53)	51
Male	224 (47)	49
**Age**		
18–25	134 (28)	19
26–35	128 (27)	24
36–45	82 (17)	20
46–55	73 (15)	16
56–65	45 (9)	11
66 or more	17 (4)	10
**Education**		
Primary school	6 (1)	49
High school	140 (29)	15
University education	333 (70)	36
**Region of residence within Brazil**		
South	314 (66)	15
Southeast	103 (22)	42
North	15 (3)	7
Northeast	19 (4)	28
Midwest	21 (4)	7
**Area of residence**		
Rural/city up to 20,000	72 (15)	16
Urban	407 (85)	84

**Table 2:** Emerging themes in response to the question, “Please justify your preference on the livestock production system chosen in the previous answer.” Questionnaire was applied between September 2014 and June 2015, *n* = 479 Brazilian participants.

Participants (*n* = 479)	Free-Range (*n* = 437 ^1^)	Confinement (*n* = 42 ^1^)	Cage-Free (*n* = 382 ^1^)	Cage (*n* = 97 ^1^)
**Themes ^2^**				
**Animal welfare**	317 (68%)	7 (15%)	280 (71%)	20 (19%)
**Production**	73 (16%)	27 (59%)	53 (13%)	69 (65%)
**Product quality**	65 (14%)	8 (17%)	57 (14%)	13 (12%)
**Environmental issues**	12 (2%)	4 (9%)	7 (2%)	4 (4%)
**Total**	**467**	**46**	**401**	**106**

^1^ Participants that chose a given system; ^2^ Number of times a given theme was mentioned by participants and the percentage it represents for each group (free-range, confinement, cage-free and cage).
